# What factors are associated with health‐related quality of life among patients with chronic musculoskeletal pain? A cross‐sectional study in primary health care

**DOI:** 10.1186/s12891-020-03914-x

**Published:** 2021-01-22

**Authors:** Kirsti Krohn Garnaes, Siv Mørkved, Øyvind Salvesen, Torgrim Tønne, Lars Furan, Gudmund Grønhaug, Ottar Vasseljen, Hege Hølmo Johannessen

**Affiliations:** 1 Department of Public Health and Nursing, Faculty of Medicine and Health Sciences, Norwegian University of Science and Technology (NTNU), P.O. Box 8905, 7491 Trondheim, Norway; 2 Department of Obstetrics and Gynaecology, St. Olavs Hospital, Trondheim University Hospital, P.O Box 3250, Trondheim, Norway; 3 Clinical Services, St. Olavs Hospital, Trondheim University Hospital, P.O Box 3250, Trondheim, Norway; 4 Tiller Physiotherapy and Manual Therapy, Ivar Lykkes veg 9, 7075 Tiller, Norway; 5 Stokmoen Physiotherapy, Wergelandsveien 27, 7504 Stjørdal, Norway; 6 Department of Health and Welfare, Østfold University College, Kobberslagerstredet 5, Fredrikstad, Norway; 7 Department of Physical Medicine and Rehabilitation, Østfold Hospital Trust, P.O. Box 300, Sarpsborg, Norway

**Keywords:** Chronic musculoskeletal pain, Health‐related quality of life, Risk factors, Multisite pain, Psychosocial factors

## Abstract

**Background:**

Chronic musculoskeletal pain (CMP) affects daily life function and is the most prevalent disorder in primary health care. The primary objective was to examine demographic factors and pain characteristics associated with reduced health-related quality of life (HRQoL) among patients in primary care reporting CMP. Our secondary objective was to compare HRQoL in patients with and without CMP.

**Method:**

This cross-sectional study was conducted in Trondheim, Norway. Twenty randomly selected GPs, and their listed patients aged 21–58 were invited to participate. Self-reported CMP data was collected using online questionnaires. HRQoL was measured by the 15D questionnaire, total score of 0.9 was used as cut-off for clinical reduced HRQoL.

**Results:**

A total of 969 patients (650 females) were recruited from six GPs’ patient lists, mean age 45.6 (SD 10.1). CMP was reported by 517 (53%). Factors significantly associated with reduced HRQoL were gender (OR 2.0, 95% CI 1.2, 3.4), disability pension (OR 26.6, 95% CI 3.1, 228.0), mood (OR 1.3, 95% CI 1.1, 1.6), relations with other people (OR 0.8, 95% CI 0.6, 0.9), sleep (OR 1.2, 95% CI 1.0, 1.3) and enjoyment (OR 1.2, 95% CI 1.0). CMP patients had significantly lower total HRQoL score compared to patients without CMP (Between group difference 0.08, 95% CI 0.07–0.09). Half of the CMP patients reported a HRQoL score < 0.9 compared to 14% in the no CMP group.

**Conclusions:**

Being female, receiving disability pension, and several psychosocial factors were found highly associated with reduced HRQoL in CMP patients, whereas pain characteristics were not. Patients with CMP reported statistically and clinically significant lower HRQoL than patients without CMP. Due to low response rate the conclusions must be handled with caution.

**Trial registration:**

Clinicaltrials.gov (NCT02020772)

## Introduction

Chronic musculoskeletal pain (CMP) is characterized by prolonged pain affecting muscles, joints or bones. CMP may be single- or multi sited, and may be present in absence of any disease [1]. CMP is very common in the general population and represents a major burden to both the individual and the society [2]. Studies have found CMP to be associated with reduced daily life function [3], disability, distress, self-perceived poor health [4], and that CMP may lead to fatigue and insomnia [5–11]. Further, CMP appears to be an independent predictor for high levels of sick leave [12, 13], and a leading cause for seeking primary health care [14], substantially contributing to inflating health care costs [14, 15]. CMP may be defined according to the ICD-11 classification system as “persistent or recurrent pain that arises as part of a disease process directly affecting bone(s), joint(s),muscle(s), or related soft tissue(s)” [16], persisting more than three months [17]. The prevalence of CMP in Europe is about 20–36% [14, 18–20]. The risk of CMP increases with age, and CMP is more prevalent among women than men [14, 21–23]. Due to an aging population, increased inactivity and obesity in the general population, the prevalence and burden of CMP is expected to rise [10]. The Norwegian general population study (the HUNT-study) [24] reported a 20% prevalence of CMP in the general population, with rising numbers during the four years follow-up. Interestingly, the tendency of increasing prevalence was most evident in the age group 20–29 years.

Chronic pain is found to be associated with reduced health-related quality of life (HRQoL) in general [25, 26], and in different patient and diagnostic subgroups [27–29]. A limited number of studies have assessed factors associated with reduced HRQoL in CMP patients. Number of pain sites and pain intensity have been highlighted as important factors affecting HRQoL, but some studies have found stronger associations with mental health factors, thus the causality of CMP remains unclear [26, 30–32]. To improve management of CMP patients in primary health care, and for clinicians to plan and offer optimal treatment, further studies are needed.

The primary aim of this study was to examine demographic and pain characteristics associated with reduced HRQoL among patients with CMP in primary health care. Our secondary aim was to compare HRQoL among patients with and without CMP.

## Methods

### Study design

This is a cross-sectional study based on data collected from patients of general practitioners (GPs) in Trondheim municipality, Norway, during the period November 2013 to July 2015.

### Subjects

Among 162 GPs in Trondheim, 20 GPs were randomly identified by using a computer random number generator, developed and administrated at the Unit for Applied Clinical Research, Norwegian University of Science and Technology (NTNU), Trondheim, Norway. The randomization of GPs was stratified by gender, to prevent gender influencing our patient selection and results. The 20 randomly selected GPs were invited to participate in the study by postal mail and telephone. To balance urbane patient populations, we included two additional GPs (male and female) from more rural areas.

The common age for pension in Norway is 67 years. All patients registered in the GPs’ patient lists, aged 21 and 58 years were invited to participate in the study. The age range 21 to 58 years was selected in order to ensure that the included patients would be from a working population throughout the three-year study period. There were no medical exclusion criteria. All eligible patients received information about the study and an invitation to participate by postal mail. Eligible patients accepted the invitation by replying to the invitational letter in writing, by phone, or by completing the web-based questionnaire (CheckWare® online Survey system, Norway). Informed consent was provided by logging into the web-based questionnaire.

### Measures

All data were collected by self-reported online questionnaires (CheckWare). The participants completed the questionnaires within one month after consenting. An automatic reminder was sent to non-responders after three weeks.

Patients responding “yes” to the question “Have you experienced pain and/or disabilities related to the muscular or the skeletal system, with continuous duration of at least 3 months during the last year?” were categorized as experiencing CMP.

### Health related quality of life

Measures developed for assessing HRQoL in patients with chronic musculoskeletal pain include the Short Form (SF)-36 Health Survey [33], the EuroQol five dimensions questionnaire [34], the World Health Organization QOL Questionnaire [35], and the 15D questionnaire [36]. In the present study we chose to assess HRQoL using the 15D questionnaire (www.15d-instrument.net) [36] as the 15D is a 15-dimensional self-reported, generic, and comprehensive questionnaire measuring HRQoL in adults aged 16 years or older [36]. The Norwegian version of the 15D questionnaire has previously been found valid and sensitive to measure quality of life among patients with chronic pain [37, 38]. The 15 dimensions include mobility, vision, hearing, breathing, sleeping, eating, speech, excretion, usual activities, mental function, discomfort and symptoms, depression, distress, vitality and sexual activity. Each dimension has five response alternatives; no problems, slight, considerable, severe, or unbearable. The total 15D scoring scale ranges from 0 representing “being dead” to 1 representing “perfect health-related quality of life” and no problems in any dimension [36]. Population based preference weights were used to generate 15D sub scores and the total 15D score (single index number). A change of 0.015 or more in 15D HRQoL score is found to be clinically relevant for self-perceived HRQoL [39]. A 15D HRQoL total score below 0.9 represents a reduced HRQoL of clinical relevance, and was used as the cut-off value for reduced HRQoL in the present study [38, 40].

### Pain related characteristics

Questions assessing pain related characteristics were designed for this study based on questions from the “SF-36 Health Survey (SF-36®/SF-36v2®, Ware JE; Sherbourne CD, 1990,1998)” [33, 41], the “Nordic musculoskeletal questionnaire” (NMQ) [42], and the “Brief Pain Inventory Long - Form (BPI)” [43]. Both the NMQ and the BPI have been tested for validity and reliability, and translated into Norwegian [44, 45]. Also the Norwegian version of “SF-36 Health Survey” is psychometrically tested and validated in a Norwegian population [46–48].

The patients who reported CMP were asked to indicate pain sites, pain intensity and how musculoskeletal pain influence general daily life function. Pain and duration of pain was assessed by the questions; 1.) “Do you have current pain which has lasted for more than 6 months?” Yes/No. 2.) “How would you rate your current pain?” 1. None, 2. Very mild, 3. Mild, 4. Moderate, 5. Severe, 6. Very severe. Patients indicated pain location(s) and number of pain sites on the NMQ digital pain map. The response alternatives for pain sites were: head, neck/shoulder, shoulder, upper back, chest, elbow, abdomen, lower back, hand, hip/thighs, knee, ankle/foot. For the statistical analyses, pain sites were grouped into six pain areas; head/neck/shoulder, chest/abdomen, elbow/hand, upper back, lower back, hip/thigh/knee/ankle/foot. Intensity of pain was assessed by patients rating the intensity of their current perceived pain on an 11-point numerical rating scale ranging from “0” (No pain), to “10” (Worst pain imaginable). For the statistical analyses current pain intensity was categorized as no to little pain (0–2), as low to moderate pain (3–5), as severe pain (6–8), and very severe pain (9–10). How pain influenced various daily life functions during the last 24 hours was evaluated by participants rating functions such as general activity, mood, walking ability, normal work, relations with other people, sleep, and enjoyment on a 11-point numerical rating scale from no influence (0) to large influence (10). A mean score was calculated for each variable. Current self-reported health was reported as either excellent, very good, good, fair or poor, and self-assessed economic status was reported as either good, fair, poor [49, 50].

### Ethics

The Regional Committee for Medical and Health Research Ethics in Central Norway (2012/1232) approved the study and study procedures followed the Helsinki Declaration. The participants signed digital informed consent prior to participation. The participants who attended the study were part of a prize draw for a gift card worth 500 NOK.

### Statistics

Participant characteristics and demographic variables are presented as mean and standard deviation (SD), or number (n) and percentage (%), as appropriate. When comparing participants with and without CMP the independent samples t-test was used for continuous variables (age), the Chi-square test was used for dichotomous variables, and the Mann-Whitney U-test for ordinal variables.

Due to a skewed distribution of 15D scores, HRQoL was dichotomized using a threshold of 0.9 in order to analyze factors associated with HRQoL using univariable and multivariable logistic regression analyses. An odds ratio (OR) above 1 represents an increased risk of reporting reduced HRQoL (total 15D score below 0.9). Statistically significant variables in the univariable analyses were included in multivariable analysis. *P*-values less than 0.05 were considered statistically significant. The statistical analyses were conducted using IBM SPSS Statistics version 25 and R version 2.13.1.

## Results

**Fig. 1:**
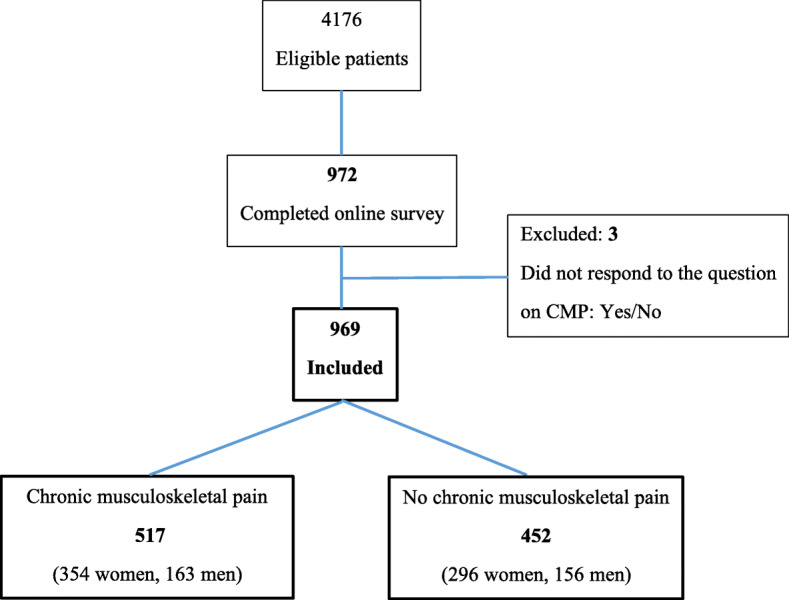
Flow-chart of study participants (*n* = 969)

Six GPs (three male and three female) agreed to participate. Among the eligible patients aged 21–58 in the GPs’ lists, 972 (23.3%) agreed to participate (Fig. 1).

### Subject characteristics and demographic variables

Just over half of the included patients (517/969) reported CMP. CMP patients were significantly older, had lower education and more worked part-time compared to patients without CMP (Table 1). Approximately one in three CMP patients reported their health to be “not so good”, compared to 8% in patients without CMP. Current sick leave due to CMP was reported by 13%, and almost half (46.3%) had been on sick leave for more than three months (Table 1).

**Table 1:** Demographic characteristics of primary health care patients grouped according to presence of chronic musculoskeletal pain (CMP) (*n* = 969)

***Patient characteristics***	***Chronic Muskuloskeletal Pain (n = 517)***	***No chronic Muskoloskeletal Pain (n = 452)***	*P*-value
	n (%)	n (%)	
***Age (years)**** mean (SD)*	45.6 (10.1)	42.6 (10.2)	˂ 0.01*
* 21–30 years*	57 (11.3)	66 (17.8)	
31–40 years	88 (17.5)	101 (23.3)	
41–50 years	156 (31.0)	134 (30.9)	
51 years and older	202 (40.2)	121 (27.9)	
*Missing*	*14 (2.7)*	*17 (3.8)*	
***Gender***			0.36**
Female	354 (68.5)	296 (65.7)	
Male	163 (31.5)	156 (34.3)	
***Marital status***			0.13***
Married/cohabitant	405 (78.6)	348 (77.5)	
Single	74 (14.4)	77 (17.1)	
Divorced/Separated/Widowed	36 (7.0)	24 (5.2)	
*Missing*	*2 (0.4)*	*3 (0.7)*	
***Education***			˂ 0.01***
Primary/secondary school	175 (34.0)	106 (23.5)	
University ˂ 4 years	141 (27.4)	137 (30.4)	
University ˃ 4 years	172 (33.4)	190 (42.1)	
Student/other	27 (5.2)	18 (4.0)	
*Missing*	*2 (0.4)*	*1 (0.2)*	
***Occupational activity***^***a***^			
None	28 (5.4)	15 (3.3)	0.12**
Full time work	358 (69.2)	348 (77.0)	˂ 0.01**
Part time work	80 (15.5)	57 (12.6)	0.23**
Under education	33 (6.4)	31 (6.9)	0.80**
Disability pension	35 (6.8)	3 (0.7)	˂ 0.001**
***Receiving benefit payments***	369 (71.4)	309 (68.4)	0.33**
***Self-assessed economic status***			0.02***
Good	273 (53.2)	277 (61.6)	
Fair	205 (40.0)	153 (34.0)	
Poor	35 (6.8)	20 (4.4)	
*Missing*	*4 (0.8)*	*2 (0.4)*	
***Self-assessed health***			˂ 0.001***
Very good	59 (11.4)	180 (40.0)	
Good	257 (49.8)	231 (51.3)	
Not so good	174 (33.7)	37 (8.2)	
Poor	26 (5.0)	2 (0.4)	
*Missing*	*1 (0.2)*	*2 (0.4)*	
***HRQoL total 15D score**** mean (SD)*	0.88 (0.10)	0.95 (0.06)	˂ 0.001*
HRQoL total 15D score < 0.9	254 (50.2)	63 (14.1)	˂ 0.001**
*Missing*	*9 (1.7)*	*6 (1.3)*	
***Current sick leave due to CMP***	69 (13.3)	-	
*Missing*	*5 (0.9)*	-	
***Duration of current sick leave***			
0–3 months	37 (53.6)	-	
3–6 months	15 (21.7)	-	
˃ 6 months	17 (24.6)	-	
*Missing*	*5 (0.9)*	-	

Numbers are presents as n (%) unless otherwise stated

*Abbreviation*: *HRQoL* Health Related Quality of Life

*Independent Samples T-Test; **Chi-squared test; ***Mann-Whitney U-test

^a^Multiple response alternatives was allowed for occupational activity

### HRQoL among patients with and without CMP

**Fig. 2:**
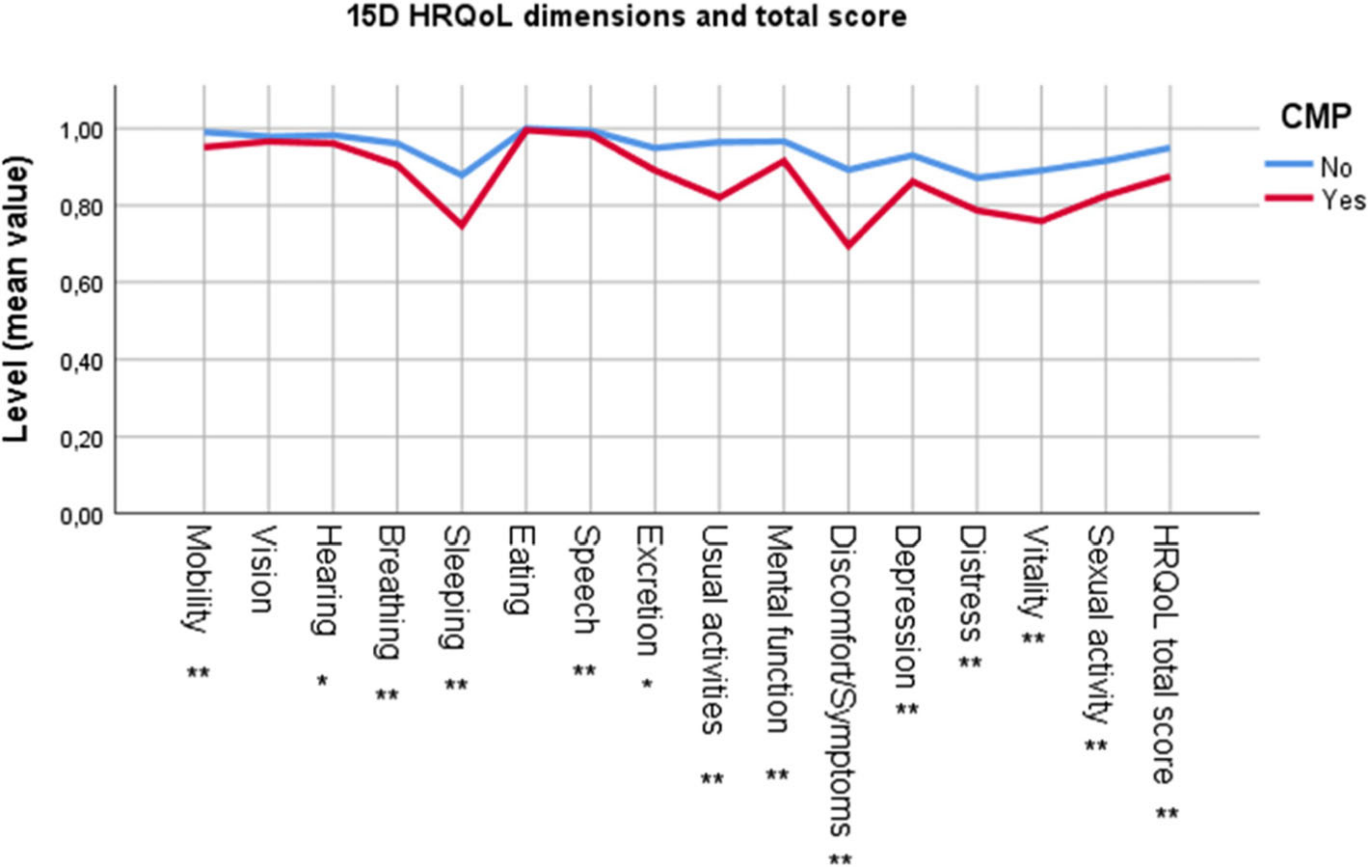
HRQoL measured by 15D HRQoL questionnaire among patients with and without CMP, presented by mean sub-score for all 15 dimensions and the mean total 15D HRQoL score

The total 15D HRQoL score and the 15D HRQoL dimension scores (15 categories) for the two groups are presented in Fig. 2. The patients with CMP reported significantly lower mean total HRQoL score compared to the patients without CMP (between group difference 0.08, 95% CI 0.07, 0.09) (Table 1). In the present study, a 15D total score < 0.9 represents a clinical reduction in HRQoL. Half of the patients with CMP reported a total score below 0.9 compared to 14% in the no CMP group. The 15D HRQoL dimension sub scores were significantly lower among patients with CMP compared to participants without CMP in most dimensions, except in the dimensions “Eating” (*p* = 0.13) and “Vision” (*p* = 0.06). The highest between-groups difference in the 15D sub scores were found in the dimensions “Discomfort/Symptoms” (CMP 0.79, SD 0.23, no CMP 0.87, SD 0.17, *p* ˂0.001) and “Sleeping” (CMP 0.75, SD 0.22, no CMP 0.88, SD 0.19, *p* ˂0.001) (Fig. 2).

### Demographic factors associated with HRQoL among patients with CMP

The univariable logistic regression analyses showed that being female, receiving disability pension, and having no occupational activity were factors significantly associated with reduced HRQoL (a total score below 0.9). Patients who had completed higher education and/or in full time employment had a significantly lower risk of reporting reductions in HRQoL. In the multivariable regression analyses, being female and receiving disability pension were the only demographic factors that remained significantly associated with reduced HRQoL (total score below 0.9), although the confidence interval for receiving disability pension was very wide (Table 2).

**Table 2:** Demographic factors and associations with reduced health-related quality of life (15D HRQoL total score < 0.9) among patients with chronic musculoskeletal pain: results from logistic regression analyses (*n* = 517)

***Demographic characteristics***	***Univariable analyses***		***Multivariable analyses***	
	*OR*	*CI*	*OR*	*CI*
***Age (years)**** (n = 503)*				
21–30 years	0.9	0.5, 1.6		
31–40 years	0.6	0.4, 1.0		
41–50 years	1.0			
51 years and older	1.2	0.8, 1.9		
***Gender**** (n = 517)*				
Female	**1.70**	**1.16, 2.49**	**2.0**	**1.2, 3.4**
Male	1.0		1.0	
***Marital status**** (n = 515)*				
Married/cohabitant	1.0			
Single	0.8	0.5, 1.3		
Divorced/Separated/widowed	1.7	0.8, 3.4		
***Education**** (n = 515)*				
Primary/secondary school	1.0			
University < 4 years	0.6	0.4, 1.0		
University > 4 years	**0.6**	**0.4, 0.9**		
Student/other	0.7	0.3, 1.5		
***Occupational activity***^***a***^* (n = 517)*				
None	**3.9**	**1.6, 9.8**	1.9	0.5, 7.0
Full time work	**0.4**	**0.2, 0.5**	0.9	0.5, 1.6
Part time work	1.3	0.8, 2.1		
Student	0.8	0.4, 1.7		
Receiving disability pension	**38.8**	**5.3, 286**	**26.6**	**3.1, 228**

Bold indicates statistically significance

*Abbreviations*: *OR* Odds ratio, *CI* Confidence interval

^a^Multiple response alternatives was allowed for occupational activity, analysed as individual variables

### Pain characteristics among patients with CMP and their associations with HRQoL

The majority of CMP patients reported three pain sites or more, and the most common pain site was the lower back. Most patients graded their current pain intensity as “low” to “moderate”, and among daily life activities, sleep and general daily activity were most affected by pain. In the univariable logistic regression analyses, almost all the pain-related variables were significantly associated with reduced HRQoL (total score below 0.9). Factors that remained significantly associated with reduced HRQoL in the multivariable analyses were everyday life functions affected by pain such as mood, relations to other people, sleep and enjoyment (Table 3).

**Table 3:** Pain characteristics among primary health care patients with chronic musculoskeletal pain (CMP) and associations with health-related quality of life: results from logistic regression analyses (*n* = 517)

***Pain-related characteristics***	***CMP patients***	***Univariable analyses***		***Multivariable analyses***	
	n (%)	OR	95% CI	OR	95% CI
***Number of pain site***^**g**^* mean (SD)*	3.5 (2.3)				
0–2	208 (40.2)	1.0		1.0	
3–5	214 (41.4)	**2.5**	**1.7, 3.8**	1.6	0.9, 2.7
6–8	73 (14.1)	**9.0**	**4.7, 17.3**	2.0	0.7, 5.7
9–12	21 (4.1)	**12.1**	**3.4, 42.9**	1.5	0.2, 10.9
***Pain sites***^a^* mean (%)*					
Head/neck/shoulder	87 (16.8)	**4.4**	**2.6, 7.6**	1.7	0.8, 3.7
Chest/abdomen	176 (34.0)	**4.5**	**1.3, 15.9**	2.9	0.6, 13.8
Elbow/hand	16 (3.1)	1.4	0.9, 2.0		
Upper back	134 (25.9)	**2.0**	**1.4, 3.1**	0.8	0.5, 1.5
Lower back	235 (45.5)	**2.1**	**1.5, 3.0**	1.3	0.8, 2.1
Hip/thigh/Knee/ankle/foot	39 (7.5)	**3.1**	**1.5, 6.6**	1.2	0.4, 4.4
***Current pain intensity***^b^* mean (SD)*					
0–2 No to little pain	238 (46.0)	1.0		1.0	
3–5 Low to moderate	195 (37.7)	**3.9**	**2.6, 5.8**	0.9	0.6, 1.0
6–8 Severe	73 (14.1)	**8.5**	**4.5, 16.1**	1.1	0.6, 13.4
9–10 Very severe to worst pain ever experienced	3 (0.6)	4.6	0.4, 51.1	0.7	0.4, 36.3
***Pain more than 6 months***^***c***^	420 (81.2)	**3.9**	**2.3, 6.4**	1.3	0.7, 2.6
***Pain influence on everyday life**** mean (SD)*					
General activity^d^	3.0 (2.6)	**1.5**	**1.4, 1.6**	1.1	0.9, 1.3
Mood^d^	2.5 (2.6)	**1.6**	**1.5, 1.8**	**1.3**	**1.1, 1.6**
Walking ability^d^	1.6 (2.4)	**1.4**	**1.2, 1.5**	1.1	0.9, 1.2
Normal working abillity ^b^	2.7 (2.8)	**1.5**	**1.3, 1.6**	1.0	0.9, 1.2
Relations with other people^b^	1.7 (2.4)	**1.6**	**1.4, 1.8**	**0.8**	**0.6, 0.9**
Sleep^e^	2.9 (2.9)	**1.5**	**1.4, 1.6**	**1.2**	**1.0, 1.3**
Enjoyment^e^	2.4 (2.6)	**1.7**	**1.5, 1.8**	**1.2**	**1.0, 1.5**

Numbers are n (%) unless otherwise stated; Bold indicates significant association between CP and pain related characteristics

*CMP* Chronic musculoskeletal pain, *SD* Standard deviation

Missing: ^a^*n* = 516; ^b^*n* = 515; ^c^*n* = 514; ^d^*n* = 513; ^e^*n* = 512; ^f^*n* = 509

^g^Multiple response alternatives were allowed for occupational activity, analysed as individual variables

## Discussion

In this cross-sectional observational study among 969 patients in primary health care practice, 53% reported CMP. Among the CMP patients, nearly half experienced lower back pain, most had low to moderate pain intensity, and more than half reported three or more pain sites. CMP patients had significantly lower total HRQoL score compared to patients without CMP, and “Discomfort/Symptoms” and “Sleeping” were the HRQoL dimensions most influenced by CMP. Half of the group of CMP patients reported a HRQoL score representing a clinically impaired quality of life. In the multivariable analyses, only gender and receiving disability pension remained significantly associated with reduced HRQoL among the demographic variables. Among the pain-related variables, the multivariable analyses showed that psychosocial factors such as impaired mood, sleep and enjoyment were significantly associated with increased risk of reporting reduced HRQoL, whereas the risk of impaired HRQoL was lower for patients who had relations with other people. Interestingly, current pain and pain sites were not associated with HRQoL in the multivariable analyses.

### Strengths and limitations

Our study is unique as it included an unselected group of patients in primary health care. Considering that, most CMP patients suffer from multisite pain, and most studies on CMP pain include single-site pain patients, the findings of our study may be highly relevant for daily clinical practice in primary health care and provide important information about the general burden of CMP. Further, the current study included a high number of participants and comprehensive information regarding pain characteristics and daily life function, which in turn gives us a valuable basis for exploring factors associated with HRQoL in this population. Assessing multiple aspects involved in the complex mechanism of CMP is vital in order to develop effective strategies to prevent and limit negative consequences of CMP at both individual and societal level. The 15-D questionnaire is a valid and reliable tool for measuring pain among primary care patients as it uses both a profile system to describe 15 dimensions important for quality of life, and an index system using population-based preferences.

There are some limitations to this study. Our study design was cross-sectional, thus we cannot imply causality or directional relationship between the different factors and HRQoL. Further, the patients themselves reported whether they fulfilled the criteria for CMP, thus the CMP categorization was mostly based on the patients’ subjective evaluation and understanding of CMP. Only one in four patients invited to the study (23%), agreed to participate and the response rate from both GPs and patients was relatively low. However, considering that approximately one in four patient in Norwegian general practice presents with musculoskeletal disorders [14], and that 53% (*n* = 517) of all invited list patients of the GP’s met the eligibility criteria for CMP, it is likely that we recruited a high proportion of patients who experienced or had experienced long-lasting musculoskeletal pain during the past 12 months. However, for background variables such as occupational activity, sick leave, prevalence of musculoskeletal disorders, disability pension/benefits the our study population is similar to the general Norwegian population in the report about work and health by the Norwegian Institute of Public Health [51]. In addition, the study population is the relatively large, thus we consider our study population to be representative for patients with CMP in primary health care. As the main focus in the present study was to explore demographic factors and pain characteristics, we did not control for confounding factors known to affect HRQoL such as anxiety, depression and physical activity. This might have introduced bias to our findings and our results must therefore be interpreted with caution.

### Comparison with other studies

Approximately half of the study population reported CMP. This is similar to a previous Danish study among 390 patients in general practice [52]. Sorensen and colleagues (2019) also found that 47% of GP patients reported musculoskeletal pain, with a duration range of 9–85 weeks [52]. In concurrence with several previous studies, we found a higher prevalence of CMP among women [3, 14, 21–23, 53].

Similar to previous findings, HRQoL was significantly lower among CMP patients compared to patients without CMP [26, 32, 54–56]. We estimated a mean difference in total HRQoL score between groups of 0.08. A difference of 0.015 or more between groups has previously been found to be clinically relevant [39]. Among the 15 dimensions included in the total 15D HRQoL score, the CMP patients in our study reported the lowest scores in the two dimensions “Sleeping” and “Discomfort/Symptoms”. The scores in the dimensions “Usual activities” and “Vitality” were also clearly lower compared to patients without CMP.

The primary aim of our study was to examine demographic factors and pain characteristics associated with HRQoL among CMP patients in primary health care practice. A limited number of studies have assessed important factors for HRQoL in this particular population. However, similar to our findings, a Norwegian cross-sectional study including a general population of chronic pain patients aged 19–81 (*n* = 1893) [26], found no association between self-reported pain and number of chronic illnesses, or global quality of health. Wahl and colleagues (2009) found that stress related symptoms, fatigue and poor self-perceived health was highly associated with lower HRQoL among CMP patients [26]. Jones and colleagues [57] addressed factors associated with HRQoL among 47 patients with chronic pain and opioid abuse. In concurrence with our findings, they reported pain intensity to be of importance for HRQoL in the univariable analyses. However, the multivariable analyses showed that pain itself was less important, while pain management, pain interference on daily life, and symptoms of depression were significantly associated with HRQoL. Malmberg-Ceder and colleagues (2016) found that psychosocial and lifestyle factors had a stronger association with work engagement than pain alone among employees with CMP [58]. Moreover, in a study among patients with chronic low back pain, high symptomatic burden, rather than perceived pain was found to be a strong predictor for reduced HRQoL [59]. In a previous study exploring predictors of quality of life among 1208 chronic pain patients, Lamè and co-workers (2004) showed that pain catastrophizing had the strongest association with quality of life in this group [60]. In the present CMP study population, multisite pain was more prevalent than single site pain, and the lower back was the most commonly reported pain site. These findings correspond to previous large studies of chronic pain [14, 61]. A higher number of pain sites and low back pain have been shown to reduce physical and work capacity [62], which in turn are associated with reduced daily life function and HRQoL [3, 60, 63]. In contrast, we found no statistically significant association between number or type of pain sites and HRQoL in our multivariate analyses, although several psychological variables related to daily life functions showed a strong association with HRQoL. Studies have shown multisite chronic pain to be associated with a major somatic symptom burden [64], which in turn may affect the patients’ physical and mental state of health, and the ability to cope with, and recover from, chronic pain [3, 65]. Studies exploring factors affecting quality of life among CMP patients indicate very complex and multifactorial associations, where daily life function and mental wellbeing seem to be of major importance for quality of life [64].

Among the demographic characteristics, we found that female CMP patients had lower HRQoL compared to male CMP patients. This is in concurrence with the study by Wahl et al. (2009), who found that female chronic pain patients had lower global quality of life than male chronic pain patients [26]. Possible reasons for this gender difference warrants further studies. Interestingly, receiving disability pension was associated with reduced HRQoL. This may be explained by assuming that these patients having endured long duration of pain, disabilities and functional disorders affecting their daily life. However, these results should be cautiously interpreted due to small sample and a large confidence interval.

### Interpretation

Even though multisite pain is common among patients with CMP, most research on CMP and HRQoL have focused on populations with single-site specific musculoskeletal pain disorders. Therefore, our study on characteristics and associations with HRQoL in an unspecified group of CMP patients in primary health care practice may provide important knowledge about the state of health and potential variables associated with quality of life. Discrepancies between reported study findings may be due to differences in pain duration and prevalence of co-existing musculoskeletal disorders. In the present study, few participants reported current sick leave due to CMP, which may indicate that our study population consisted of many patients with a relatively low symptom burden or that they were able to manage work despite pain. Inclusion criteria for CMP in this study was self-reported pain and/or disabilities related to the musculoskeletal system for 3 months or longer during the last year, indicating the patients were suffering or had suffered a recent chronic pain condition. Enhancing the knowledge base on CMP is of major importance in order to develop more precisely targeted prevention and treatment strategies in the primary health care system for this group of patients. Our findings may be useful for primary health care personnel, especially GPs and physiotherapists in clinical practice, by increasing their awareness of pain rarely being a single important factor for quality of life in this population.

### Generalisability

All participants included in our study were informed of the main focus; musculoskeletal pain. We included a high number of patients in primary health care with previous and present symptoms related to the musculoskeletal system, also among patients in the group without CMP. Our population consisted of patients receiving ongoing treatment in primary health care, and patients on the GPs’ patient lists currently receiving no active treatment. There were more females in the CMP group, and participants were slightly older, had lower education and more worked part-time compared to the group without CMP. In addition, receiving disability pension was more frequent, and self-assessed economic status and health status tended to be lower among CMP patients. These characteristics of CMP patients, in addition to multisite pain and low back pain being the most prevalent pain site, are in concurrence with findings in previous studies, and indicate that our study population may be representative of CMP patients in general [3, 56, 60, 66]. Beside age between 21 and 58 years, there were no further inclusion criteria in our study, and we targeted all CMP patients in primary health care practice. Thus, our findings may be generalizable to the general population of CMP patients with typical sociodemographic characteristics and general CMP burden.

## Conclusions

Just over half of the recruited primary health care patients reported CMP. Being female, receiving disability pension and psychosocial factors such as mood, sleep, enjoyment and relation to other people were identified as factors strongly associated with reduced HRQoL in CMP patients. Pain intensity, pain sites and type of pain sites were not significantly associated with reduced HRQoL. CMP patients reported significantly lower HRQoL than patients without CMP, and the total score indicate reduced quality of life of clinical importance for everyday living among the majority of CMP patients. Because of the relatively low response rate in our study, the conclusions must be handled with caution.In primary health care practice, it may be important to establish strategies aiming to improve psychological well-being among CMP patients and to be aware of how possible gender differences may affect quality of life in CMP patients.

## Data Availability

The datasets used and/or analyzed during the current study are available from the corresponding author on reasonable request.

## Abbreviations

CMP: Chronic musculoskeletal pain
HRQoL: Health related quality of life
GP: General practitioners
